# Non-infected penile prosthesis cultures during revision surgery; comparison between antibiotic coated and non - coated devices

**DOI:** 10.1590/S1677-5538.IBJU.2016.0061

**Published:** 2016

**Authors:** Seyfettin Ciftci, Tijen Nemut, Mustafa Melih Culha, Hasan Yilmaz, Murat Ustuner, Ufuk Yavuz, Levend Ozkan, Aynur Karadenizli, Sadi Turkan

**Affiliations:** 1 Department of Urology, University of Kocaeli Medical Faculty, Kocaeli, Turkey; 2 Department of Health School, University of Sakarya, Sakarya, Turkey; 3 Department of Urology, Derince Research and Training Hospital, Kocaeli, Turkey; 4 Department of Urology, Karaman State Hospital, Karaman, Turkey; 5 Department of Microbiology, University of Kocaeli Medical Faculty, Kocaeli, Turkey; 6 Department of Urology, Kastamonu Anadolu Hospital, Kastamonu, Turkey

**Keywords:** Penile Prosthesis, Culture, Antibiotic Prophylaxis

## Abstract

**Introduction:**

Aim of this study is to investigate bacterial growth on non-infected devices and compare antibiotic-coated and non-coated implants.

**Materials and methods:**

The charts of 71 patients who underwent revision surgeries for penile prosthesis between 1995 and 2013 were reviewed. Of those, 31 devices were antibiotic-coated prostheses, while 40 of the implants were non-coated. Swab cultures were routinely obtained from corporal, pump or reservoir site during the operation. If a bacterial biofilm was determined on the prosthesis, it was also cultured.

**Results:**

A total of 5 different organisms were cultured from 18 patients. Of them, 4 devices were antibiotic-coated and the other 14 were non-coated devices. Staphylococcus epidermidis was the most common organism, while Staphylococcus hominis, beta hemolitic streptococcus, Escherichia coli and Proteus mirabilis were also cultured. All patients who had positive cultures were treated with appropriate antibiotics for four weeks postoperatively. Median follow-up time was 41 months, ranging between 8 and 82 months. One prosthesis (non-coated) became clinically infected in the follow-up period with a totally different organism. Culture positivity rates of antibiotic-coated and non-coated devices were 13% and 35% respectively and the result was significant (p=0.00254).

**Conclusions:**

Positive bacterial cultures are present on non-infected penile prostheses at revision surgeries in some of the patients. Antibiotic coated prostheses have much less positive cultures than non-coated devices.

## INTRODUCTION

Penile implants have been used for more than 40 years and are the standard of care for erectile dysfunction in patients who do not respond to medical treatment. The morbidity rate associated with penile implants is relatively low, and patient satisfaction is high owing to improvements in techniques, materials and design. Mechanical failure, which is the primary cause of reoperations following penile prosthetic surgery, has an incidence ranging from 0–56% in large series (1–4). The most common cause of mechanical failure is fluid leakage from the device (5). Other reasons of revision include corporal deformity, aneurismal dilatation of the cylinders, lateral extrusion and the desire of the patient for upsizing (5). Infection represents one of the most serious complications of penile prosthesis implantation surgeries. The prevalence of PPIs in modern series is estimated to be between 1% and 3% for first (virgin) implantation and between 10% and 18% for repeat implantations (6, 7). Despite careful work and the use of copious antibiotic irrigation of the implant space and systemic prophylactic antibiotics, the incidence of infection ranges between 1% and 6.5% (1, 8). These rates may further decline by 50% with the use of drug-coated prostheses for both first-time and repeat implantations (9, 10). Although infection rates are low, morbidity due to infection is serious. Additionally, infection may result in corporal fibrosis and penile shortening. These complications are responsible for patient dissatisfaction and can render re-implantation difficult. An immediate salvage procedure with substantial success is used to prevent corporal fibrosis and penile shortening (11, 12).

Treatment of PPIs is difficult and costly, and the prevention of infections is accordingly of utmost importance. In the last 10 years, there have been some technical and device-related modifications that have been adopted to reduce the risk of infection associated with penile prosthesis surgery (13). After 2001, manufacturers began to coat penile implants with an antibiotic impregnated material in an attempt to protect the devices from revision surgery because of infection. However, the etiology of late prosthesis infection is still controversial. The effect of hematogenous seeding has been discussed by many authors but not proven yet. It has been shown that, the majority of clinically non-infected penile prostheses possess organisms growing in the implant space that were cultured during the revision surgery (14, 15). The bacteria are inoculated into the implant space during the implantation surgery because of local hematoma, edema, and necrosis. These bacteria secrete extracellular polysaccharide matrix called biofilm which enhances bacterial growth by acting as physical barrier to protect the bacteria from antibiotics and host defense mechanisms (15, 16). These bacteria remain dormant in the implant space and do not cause clinical infection for many years until they become planktonic (i.e., free-floating). At that time, the symptoms of an active infection are manifested. The effect of microorganisms that were cultured in revision surgeries has not yet been investigated.

In this study, we aimed to investigate bacterial growth on non-infected penile prostheses. We also compared antibiotic-coated and non-coated implants during the revision surgery.

## MATERIALS AND METHODS

This study was a retrospective evaluation of prospectively followed patients who underwent penile prosthesis revision surgery due to non-infected causes. We prospectively obtained cultures in a standard manner from patients who underwent either revision or explantation of clinically non-infected penile prostheses. We focused on a total of 71 consecutive patients who underwent revisions for clinically non-infected reasons between 1995 and 2013. The patients underwent revision surgery for mechanical failure in 57 cases (80.2%), cylinder aneurysms in 3 cases (4.2%), impending erosion of the device in 4 cases (5.6%), patient dissatisfaction in 4 cases (5.6%), and undersizing in 3 cases (4.2%). There was no evidence of clinical infection in any patients prior to the revisions, and all of the patients had negative urine cultures. Thirty-one of the devices were coated with antibiotic, while 40 were non-coated. Swab cultures were routinely obtained from the corporal, pump or reservoir site during the operation. The cultures were obtained from all of the accessible components after removal using swabs of the surface of the device and peri-prosthetic space. If a biofilm was detected on any part of the prosthesis, an additional swab culture was obtained from that area. The collected samples were enclosed in air-tight plastic tubing and then transported to the microbiology test laboratory. The swabs were inoculated on blood sheep agar and EMB agar plates and incubated for 48 hours. The agar plates were examined after 24 and 48 hours. Bacteria exhibiting growth were identified using standard techniques based on morphological, cultural and biochemical characteristics. Antimicrobial sensitivities were determined using the standard disc diffusion technique. Bacterial colony counts of the identified organisms were quantitated by our microbiology laboratory and reported.

All of the surgical procedures were performed by a single surgeon (MMC) according to standard surgical protocols (i.e., a penoscrotal incision). There were no complications during any of the surgeries. On the day of the revision surgery, all of the patients underwent a 10-minute skin preparation procedure with a povidone-iodine scrub. Furthermore, all of the patients received perioperative intravenous antibiotics (a combination of amikacin 500mg and teicoplanin 400mg). During the procedure, antibiotic irrigation was routinely performed on the surgical sites. A spray solution containing antibiotics was only used on the non-coated devices. All of the patients continued to receive intravenous antibiotics postoperatively until discharge. Fluoroquinolones were also prescribed for 7 days after discharge. All of the patients who had positive cultures were treated with appropriate antibiotics for four weeks postoperatively. After the revision surgery, urine and blood cultures were obtained from the symptomatic patients to evaluate the incidence of postoperative infections.

Different types of three-piece hydraulic prostheses were evaluated in this study. Either AMS (American Medical Systems, Minnetonka, Minnesota, USA) or Coloplast (Mentor Corporation, Santa Barbara, California, USA) penile prostheses were used in all of the patients. The prostheses not coated with antibiotics included AMS-CX, AMS-Ultrex and Mentor Alpha devices; the antibiotic-coated prostheses included AMS-CX and AMS-LGX-devices. The non-coated implants were old devices; they were used prior to 2000 and did not absorb antibiotics.

All of the data were collected from the hospital record system, and the study was performed in accordance with the ethical standards laid down in the 1964 Declaration of Helsinki and its later amendments.

### Statistical analysis

All of the collected data were entered and analyzed using the SPSS 17.0 statistical program (SPSS Inc., Chicago, IL, USA). We used an independent sample t-test and Fisher's exact tests to compare the groups. Statistical significance was set at a p value of <0.05.

## RESULTS

A comparison between the antibiotic-coated and non-coated groups is listed in Table-1. The mean ages of the patients in both groups were similar, as shown in Table-1. There were 9 and 11 patients with diabetes mellitus in the antibiotic-coated and non-coated groups, respectively; this difference was not significant (p>0.05). A total of 5 different organisms were cultured from 18 patients. Of these 18 devices, 4 were antibiotic coated and the other 14 were non-coated. Staphylococcus epidermidis was the most common organism; Staphylococcus hominis, beta hemolitic streptococcus, Escherichia coli and Proteus mirabilis were also cultured. Two different organisms were cultured simultaneously in two different patients in the antibiotic non-coated group and in one patient in the antibiotic coated group. A biofilm was detected in two patients, and the parts of the devices were also cultured. Staphylococcus epidermidis was cultured from both biofilms. The numbers of cultured organisms from both groups are listed in Table-2. The mean follow-up time was 41 months, with a range of 8–82 months. One prosthesis (non-coated) became clinically infected 17 months after the first revision surgery with a completely different organism (E. coli S. epidermidis). We cultured E. Coli from the non-infected prosthesis in the revision surgery. Later, the same prosthesis became clinically infected with S. epidermidis. The positive cultures rates of the antibiotic-coated and non-coated devices were 13% and 35%, respectively, and the difference was statistically significant (p=0.002). We found that antibiotic-coated prostheses had a culture positivity rate that was 62.9% lower than that of the non-coated group.

**Table 1:** Comparison of characteristics of both groups.

	Antibiotic coated (n=31)	Antibiotic non-coated (n=40)	p
Mean age (years)	48.5	51.2	>0.05
Positive culture n (%)	4 (13)	14 (35)	0.002
Median follow-up (months)	29 (8 to 38)	53 (14 to 82)	<0.05
Mean revision time after first implant (years)	7.1 (2.4 to 8)	9.4 (0.8 to 17)	<0.05

**Table 2:** Organisms cultured from uninfected penile prostheses during the revision surgery.

	Antibiotic coated (n=31)		Antibiotic non-coated (n=40)	
	Cultured from one component	Cultured from more than one component	Cultured from one component	Cultured from more than one component
Staphylococcus	2	1	6	3
Epidermidis				
Escherichia Coli	1	-	2	1
Beta hemolytic streptococcus	1	-	-	-
Staphylococcus Hominis	-	-	2	-
Proteus Mirabilis	-	-	2	-
None		27		26

Within the antibiotic non-coated group, 20 patients had AMS-CX devices, 8 patients had AMS-Ultrex devices, and 12 patients had Mentor Alpha devices; in the antibiotic-coated group, 26 patients had AMS-CX devices and 5 patients had AMS-LGX devices.

## DISCUSSION

While design modifications have decreased the incidence of mechanical failure, prosthesis infection remains a significant complication of penile implants. The incidence of PPIs associated with primary implantation is estimated to be between 1% and 3% for first implantation; the incidence of infection is higher after reoperation for revision or re-implantation (6, 7). Because treatment of these infections typically requires removal of the infected penile implant, preventing infections is crucial. It has been estimated that the majority of PPIs occur within the first year of implantation; the highest rate was noted within the first 3–6 months (17). In contrast, the mean revision time after implantation was 7.1 years in the antibiotic-coated group and 9.4 years in the non-coated group in our study Table-1. We believe that these long periods were due to lack of infection for revision surgeries.

Despite reducing the risk of infection with preoperative skin preparations, perioperative prophylactic antibiotics, copious irrigation with antibiotics and minimizing device-skin contact, the bacteria remains dormant on implants and may result in late prosthesis infection at later times. If revision of the device is necessary, the sequestered bacteria may be released and result in increased risks of infection in the new device (18). This situation may also lead to increased infection rates in revised penile prostheses.

Clinically apparent infections are related to the virulent species of bacteria and the host defense mechanisms. Patients with diabetes mellitus are more susceptible to developing infections as a result of high glucose levels that encourage bacterial growth. However, the number of patients with diabetes mellitus was not different significantly between our two study groups.

It has been shown that antibiotic-coated penile prostheses are associated with a lower risk of infection than non-coated devices. A recent study reported that antibiotic impregnated devices have a 1.62% infection-related revision rate; non-impregnated penile prostheses have a 4.24% infection-related revision rate at a 7 year follow-up. This difference was statistically significant (19). The authors concluded that the use of antibiotic-impregnated devices can decrease revision due to infection. Similarly, Wilson et al. found that antibiotic-coated penile prostheses resulted in fewer infections in virgin, non-diabetic and virgin, diabetic patients than non-coated prostheses (20). Controversially, these authors also found that antibiotic-coated devices used in revision surgery did not reduce the infection rate if the antibiotic wash-out was not applied. Carson et al. (17) compared infection rates between penile prostheses impregnated with InhibiZone and prostheses without antibiotic. They found that prostheses with InhibiZone had significantly lower infection rates than non-coated prostheses at all of the time points that they evaluated. The decrease in infection rates between the groups was the largest during the first 60 days of implantation (82.4%). After 180 days, the decrease fell to 57.8%, but it maintained a constant level thereafter. Carson et al. concluded that antibiotic-coated group exhibited a 56.5% decrease in infection rate compared with the non-coated group after 1 year of surgery. Although Carson et al. studied a different population than our cohort, we similarly found that antibiotic-coated prostheses had a culture positivity rate that was 62.9% lower than that of the non-coated group after a long follow-up period.

Bacteria are also cultured from the penile prostheses that were explanted during a revision surgery owing to non-infected causes. Licht et al. obtained cultures from 65 clinically non-infected penile prostheses and 22 artificial urinary sphincters during revision. These authors found that 43% of clinically non-infected penile prostheses (28 out of 65) exhibited a positive culture during revision surgery (21). Staphylococcus epidermidis was the most common isolated organism and was found in 26 out of 65 penile prostheses (40%). Licht et al. also found that 3 penile prostheses became infected during the follow-up period. However, this study was moderately early and published at the time when non-coated devices were being used commonly. In a more recent multicentric study spanning three institutions, penile prostheses cultures were obtained from 77 clinically non-infected penile prostheses during the revision surgeries (15). Microorganisms were cultured from 54 out of the 77 devices (70%). The authors also found that 49 out of 54 patients (90%) had positive culture for Staphylococcus genus; there were 10 different species. They additionally evaluated the sensitivity of organisms to rifampin and tetracycline (minocycline) and concluded that all of the staphylococcal species were sensitive to these antibiotics. Licht et al. focused on only non-coated penile prostheses and did not make a comparison between antibiotic-coated and non-coated devices. In an experimental study, Rajpurkar et al. (22) evaluated the effect of coating the surface of polyurethane (Bioflex) with a hydrophilic material with and without antibiotics. These authors quantified bacterial colony counts both in vitro and in rats. They found that bacterial colonization was the highest in the uncoated Bioflex material, followed by the uncoated Bioflex with the antibiotic treatment, coated Bioflex and coated Bioflex with antibiotic treatment. They concluded that coated Bioflex with antibiotic treatment resulted in a 55% reduction in bacterial count compared with uncoated Bioflex. Moreover, they also found that coated Bioflex without antibiotic treatment correspondeds to a 41% reduction in bacterial colonization compared with uncoated Bioflex without antibiotic treatment. The findings of this experimental study support our results that antibiotic-coated materials experience less bacterial colonization than non-coated materials. This conclusion may lead to a decreased late infection rate in antibiotic-coated penile prostheses. A study by Abouassaly et al. recovered findings similar to ours (23). The authors performed 55 replacements of penile prostheses due to reasons independent of infections; no salvage procedures were performed. Abouassaly et al. found only one case of clinical infection with a median follow-up duration of 32 months. They concluded that antibiotic-coated penile prostheses may decrease infection rates in patients undergoing replacement procedures owing to causes other than infection.

Skin flora are the most common source of PPIs; those organisms are introduced at the time of device implantation. Staphylococcus epidermidis is the most common organism cultured in infected prostheses, and it accounts for 35–80% of all positive cultures (8, 24). Parsons et al. also showed that Staphylococcus epidermidis was the most common organism cultured from infected prostheses and urinary sphincters (25). Similarly, Staphylococcus epidermidis was the most common isolated organism from revised penile implants that were revised owing to causes other than infection (15, 21). These organisms are of low virulence and are accessed during the implantation and survive in a biofilm secreted by the bacteria themselves. Our results confirmed that Staphylococcus epidermidis is the most common cultured organism in uninfected, revised penile prostheses. Staphylococcus aureus and Escherichia coli have also been shown to play a role in PPI (21, 26, 27). We additionally cultured Staphylococcus hominis, beta hemolytic streptococcus and Proteus mirabilis from the non-infected devices.

This study had a few limitations. First, although the patients were followed prospectively, the data were collected retrospectively from hospital records. Second, there have been no similar and comparable studies in the literature with a large volume of data pertaining to non-infected penile prosthesis revision surgery and comparing antibiotic-coated and non-coated devices. Despite these limitations, this study for the first time clearly showed that antibiotic-coated devices result in less bacteria colonization on the implant and peri-prosthetic space compared with non-coated devices, a situation that may decrease the incidence of PPIs.

## CONCLUSIONS

Based on our results, positive bacterial cultures are present on non-infected penile prostheses during the revision surgeries of some patients. Our results also suggest that, antibiotic-coated penile prostheses have less bacteria seeding on prostheses in revision surgeries. Therefore, antibiotic coating may prevent late PPIs, particularly after revision surgeries. We also confirmed that, Staphylococcus epidermidis was the most common cultured organism from uninfected penile prostheses during revisions.
